# Dr. John A. Hoffmeyer

**Published:** 1917-03

**Authors:** 


**Dr. John A. Hoffmeyer,** Buffalo 1880, died at his home in Black Rock, Buffalo, Jan. 25, aged 63, of heart disease. He was born and spent all his life in Black Rock.

